# Circulating Cell Free DNA as the Diagnostic Marker for Ovarian Cancer: A Systematic Review and Meta-Analysis

**DOI:** 10.1371/journal.pone.0155495

**Published:** 2016-06-02

**Authors:** Quan Zhou, Wei Li, Bingjie Leng, Wenfei Zheng, Ze He, Manzhen Zuo, Aihua Chen

**Affiliations:** Department of Gynecology and Obstetrics, the People’s Hospital of Three Gorges University/the First People’s Hospital of Yichang, Yichang 443000, China; The Ohio State University, UNITED STATES

## Abstract

**Background:**

Quantitative analyses of circulating cell-free DNA (cfDNA) are potential methods for the detection of ovarian cancer. Many studies have evaluated these approaches, but the results were too inconsistent to be conclusive. This study is the first to systematically evaluate the accuracy of circulating cfDNA for the diagnosis of ovarian cancer by conducting meta-analysis.

**Methods:**

We searched PubMed, Embase, Cochrane Library and the Chinese National Knowledge Infrastructure (CNKI) databases systematically for relevant literatures up to December 10, 2015. All analyses were conducted using Meta-DiSc1.4 and Stata 12.0 software. Sensitivity, specificity and other measures of accuracy of circulating cfDNA for the diagnosis of ovarian cancer were pooled. Meta-regression was performed to identify the sources of heterogeneity.

**Results:**

This meta-analysis included a total of 9 studies, including 462 ovarian cancer patients and 407 controls. The summary estimates for quantitative analysis of circulating cfDNA in ovarian cancer screen were as follows: sensitivity, 0.70 (95% confidence interval (CI), 0.65–0.74); specificity, 0.90 (95% CI, 0.87–0.93); positive likelihood ratio, 6.60 (95% CI, 3.90–11.17); negative likelihood ratio, 0.34 (95% CI, 0.25–0.47); diagnostic odds ratio, 26.05 (95% CI, 14.67–46.26); and area under the curve, 0.89 (95% CI, 0.83–0.95), respectively. There was no statistical significance for the evaluation of publication bias.

**Conclusions:**

Current evidence suggests that quantitative analysis of cfDNA has unsatisfactory sensitivity but acceptable specificity for the diagnosis of ovarian cancer. Further large-scale prospective studies are required to validate the potential applicability of using circulating cfDNA alone or in combination with conventional markers as diagnostic biomarker for ovarian cancer and explore potential factors that may influence the accuracy of ovarian cancer diagnosis.

## Introduction

Cancer constitutes an enormous burden on society in developed and developing countries alike [1]. Ovarian cancer is the most lethal form of all gynecological malignancies and the fifth most common cause of cancer death in women [2]. Each year, more than 230,000 new cases are diagnosed and 151,900 women died from ovarian cancer in worldwide [1]. Its lethality may be due to the lack of specific symptoms and effective screening and early diagnostic methods to detect the disease. Over 75% of patients are at advanced stage of the disease (Stage III or IV) when being diagnosed, with only 5%-21% of 10-year survival rate [3]. Therefore, the development of sensitive and specific diagnostic methods or biomarkers for early detection of ovarian cancer is urgently needed.

Currently, histopathology examination is considered the gold standard for ovarian cancer diagnosis, but it is time consuming, costly and difficult to obtain tumor samples, which limits its application in early diagnosis. Thus, bimanual pelvic examination, cancer antigen (CA) 125 and transvaginal sonography are widely employed as main diagnostic tools for early diagnosis of ovarian cancer [3–5]. Unfortunately, several high-quality studies have demonstrated that the bimanual pelvic examination lacks accuracy as a screening method for ovarian cancer [6–8]. CA125, a tumor-specific antigen, is frequently used to detect ovarian cancer and is elevated in 80% of women with advanced ovarian carcinomas [3]. However, it has low diagnostic sensitivity (50%-62% for early stage ovarian cancer) and limited specificity (73%-77%) [4, 9]. Transvaginal sonography is a useful preoperative examination for predicting the diagnosis of pelvic masses, but it requires a specific device and its diagnostic accuracy is largely affected by the experience of the examiner [10]. Therefore, minimally invasive and highly accurate diagnostic methods for detection of ovarian cancer are promptly needed, so as to better improve the prognosis of patients with the disease.

Circulating cell-free DNA (cfDNA) is a type of cell-free nucleic acids that is released by both normal and tumor cells into the circulation through cellular necrosis and apoptosis [11]. Recently, some studies report that quantitative analysis of circulating cfDNA is an emerging non-invasive blood biomarker that can be utilized to assess tumor progression and predict prognosis, diagnosis and response to treatment in several types of cancers including ovarian cancer [12–14]. In particular, significantly elevated cfDNA levels have been detected in ovarian cancer patients, compared with healthy subjects [15–24].Thus, the quantitative assay of plasma DNA has been proposed as a screening tool for ovarian cancer [16–24]. A good number of studies have reported the potential of using circulating cfDNA as a novel diagnostic marker for ovarian cancer [16–24]. However, many of the published studies contain inconsistent results, and there are not any previous meta-analyses in the literature which covered this research question. In the present study, we conducted the meta-analysis using data from multiple studies to systematically evaluate the potential of using circulating cfDNA as non-invasive biomarkers in the diagnosis of ovarian cancer.

## Materials and Methods

### Search strategy

The meta-analysis was conducted following the criteria of Preferred Reporting Items for Systematic Reviews and Meta Analyses (PRISMA) [25] (S1 Appendix). A comprehensive literature search was performed using PubMed, Embase, Cochrane Library and Chinese National Knowledge Infrastructure (CNKI) databases for all relevant articles without language limitation. No limitation was set on the start date for the publications, and the search ended on December 10, 2015. The following retrieval indexes were used: (("Ovarian Neoplasms/diagnosis"[Mesh]) OR ‘ovarian neoplasms’ OR ‘ovarian carcinoma’ OR ‘ovarian tumor’ OR ‘ovarian cancer’) AND (‘cell free DNA’ OR ‘circulating DNA’ OR ‘cfDNA’) AND (‘blood’ OR ‘serum’ OR ‘plasma’ or ‘circulation’) AND (‘diagnoses OR ‘sensitivity and specificity’ OR ‘ROC curve’). In addition, reference lists of the included articles were cross-checked to search for additional relevant studies that were not detected by the original literature search.

### Inclusion and exclusion criteria

The inclusion criteria for the publications are as follows: (1) evaluated diagnostic accuracy of quantitative analysis of circulating cfDNA for ovarian cancer; (2) sensitivity and specificity were reported or could be calculated from 2×2 contingency tables; (3) absolute numbers of true-positive (TP), false-positive (FP), true-negative (TN), and false-negative (FN) cases were provided; (4) full data set could be retrieved from the publication and the full-text article was available; (5) only studies that include at least 10 ovarian cancer patients were selected since very small sample size may lead to selection bias.

Studies with the following characteristics were excluded: (1) Studies with incomplete data, data that could not be retrieved or reconstructed for 2×2 tables; (2) Studies that overlapped the included studies (i.e., studies from the same study group, institution, and with the same results); (3) Unsuitable publication types, including comments, letters, editorials and expert opinions, reviews without original data, case reports or studies with fewer than 10 patients. Two reviewers (Q Zhou and BJ Leng) independently determined the eligibility of the studies, and disagreements in decisions were resolved by consensus. When the same patient population was used in several studies, only the most recent, largest or best-quality study was included.

### Data retrieval

Two reviewers (Q Zhou and BJ Leng) independently retrieved data from all eligible studies. The data included: (1) basic characteristics of studies, including last name of the first author, year of publication, country of origin, sample size, methods of detection, type of specimens; (2) diagnostic performance, including sensitivity, specificity, TP, FP, TN, and FN. The reviewers were blinded to publication details, and disagreements between them were resolved by consensus.

### Quality assessment

Bias is a systematic error, or deviation from the truth, in results or inferences and include selection bias, performance bias, attrition bias, detection bias and reporting bias, To assess the methodological quality of each study and potential risk of bias, we used the Quality Assessment of Diagnostic Accuracy Studies-2 (QUADAS-2) tool [26]. The QUADAS-2 tool is comprised of four key domains: patient selection, index test, reference standard, and flow and timing. We used seven items from the QUADAS-2 to evaluate the quality of included studies. Each of which was answered as ‘‘yes”, ‘‘no”, or ‘‘unclear”. An answer of “yes” means low risk of bias, while an answer of ‘‘no” or ‘‘unclear” indicates high risk of bias. If a study is judged as ‘‘low” on all domains relating to bias or applicability, it is appropriate to have an overall judgment of ‘‘low risk of bias” or “low concern regarding applicability” for that study. If a study is judged ‘‘high” or ‘‘unclear” in one or more domains, then it may be judged ‘‘at risk of bias” or as “having concerns regarding applicability”. Quality assessment of the included studies was performed and cross-checked independently by two reviewers. In case of conflict, a third reviewer was consulted, and disagreement was settled through multilateral discussion.

### Statistical analysis

We used standard methods recommended for meta-analysis of diagnostic test evaluations [27–29]. Statistical analysis was performed utilizing Meta-DiSc 1.4 (Cochrane Colloquium, Barcelona, Spain) and Stata 12.0 (Stata Corporation, College Station, USA) software. The bivariate meta-analysis model was employed to summarize the sensitivity, specificity, diagnostic odds ratio (DOR), positive likelihood ratio (PLR), and negative likelihood ratio (NLR). Meanwhile, the bivariate SROC and its 95% confidence interval (95% CI) were generated by plotting the sensitivity and specificity of each of the included studies [30]. The area under the curve (AUC) was used for grading the overall accuracy as a potential summary of the SROC curve [31]. In addition, the threshold effect was detected by the Spearman correlation coefficient (between the logit of sensitivity and logit of 1-specificity), a value of *P less than* 0.05 indicated significant threshold effect. The chi-square and *I*^*2*^ test were used to assess the heterogeneity between studies. A value of *P* less than 0.1 or an *I*^*2*^ higher than 50% indicated the existence of significant heterogeneity [32, 33]. Subgroup analysis and meta-regression analyses were performed to explore the potential sources of between-study heterogeneity. Deeks’ funnel plot asymmetry test was performed for DOR to explore the possibility of publication bias, among studies, and *P*<0.01 was considered representative of significant statistical publication bias [34]. All statistical tests were two-sided, and a *P*<0.05 was considered statistically significant.

## Results and Discussion

### Search results

**Fig 1:**
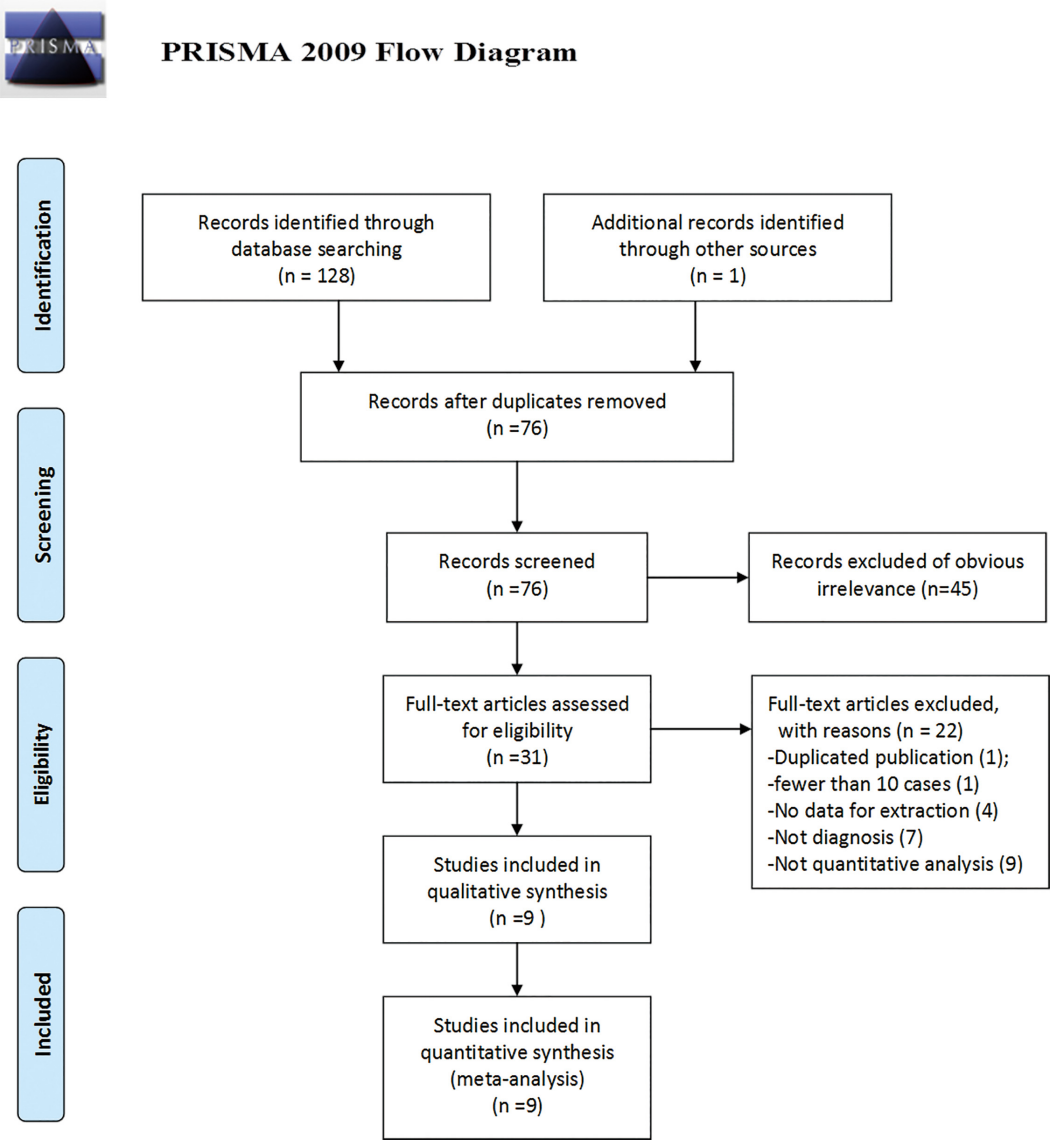
Flow chart showing the process for selecting eligible studies in the meta-analysis.

The initial search retrieved a total of 129 publications (128 through a database search and 1 through other sources). After removal of duplicates, we obtained 76 publications. The titles, abstracts, and key words were then carefully evaluated, and 45 studies were excluded (12 studies were excluded due to unsuitable publication types including reviews and commentaries, 22 studies were excluded as non-diagnostic studies, and 11 were excluded for focusing on carcinomas other than ovarian cancer). Afterward, the remaining 31 articles were subjected to full text review and 22 articles were excluded (one study had significant overlap (the same author, the same institution)) with another study which had better quality, one study with fewer than 10 cases, seven studies were not related to diagnosis, four lacked necessary data and nine were not a quantitative analysis of circulating cfDNA for the diagnosis of ovarian cancer. Consequently, we obtained 9 publications [16–24] that met all of the inclusion criteria and none of the exclusion criteria for meta-analysis. The flowchart for inclusion and exclusion of the studies is presented in Fig 1.

### Characteristics of included studies and quality assessments

In this meta-analysis, the final set of 9 diagnostic studies [16–24] included a total of 462 patients with ovarian cancer and 407 healthy control individuals. All the ovarian cancer patients were diagnosed based on histopathological examination. Regarding the origin of the studies, four studies [18–20, 23] were conducted in Asia (China), two in the USA [17, 22], and three in Europe (Italy, Germany and Switzerland) [16, 21, 24]. All studies were published from 2001 to 2014, and the number of ovarian cancer patients in each study varied from 21 to 93. Several different methods were used in these studies to measure the quantity of circulating cfDNA: five studies [16, 18, 20, 22, 24] used quantitative real-time polymerase chain reaction (RT-PCR), two studies [17, 19] performed fluorescence staining (PicoGreen and SYBR GreenI), one study conducted ELISA [21] and one study used branched DNA (bDNA) [23]. In terms of diagnostic accuracy of cfDNA in ovarian cancer, six studies [16–18, 20, 22, 24] used plasma cfDNA, and the remaining three studies [19, 21, 23] used serum cfDNA. For data collection, only one study had a prospective design [16] and another study had a retrospective design [17]. Most studies did not report how they collected data. The main features of the included studies are described in Table 1.

**Table 1:** Summary of Studies Included in the Meta-Analysis.

Study	Country	Year	No. ofP/C	Assaymethod	Study design	Tumorstage	Tumor grade	Sample	Cutoff	TP	FP	FN	TN	Sensitivity(%)	Specificity(%)
Capizzi[16]	Italy	2008	22/50	RT-PCR	prospective	IIIC-IV	G1-G3	plasma	14.5ng/mL	17	2	5	48	77.0	96.0
Chang[17]	USA	2002	54/30	PicoGreen	retrospective	I-IV	NR	plasma	60.0ng/mL	29	0	25	30	54.0	100
Fu[18]	China	2005	93/100	RT-PCR	NR	I-IV	G1-G3	plasma	44.0μg /L	61	9	32	91	65.6	90.9
Gu[19]	China	2009	30/15	SYBR GreenI	NR	I-IV	G1-G3	serum	33.0ng /mL	13	1	17	14	43.3	93.3
Guan[20]	China	2008	30/20	RT-PCR	NR	I-IV	G1-G3	plasma	15.7μg /L	19	2	11	18	63.3	90.0
Holdenrieder[21]	Germany	2001	63/45	ELISA	NR	NR	NR	serum	216.0AU	23	0	22	63	51.1	100
Kamat[22]	USA	2010	82/62	RT-PCR	NR	I-IV	G1-G3	plasma	4500GE/ml	75	9	7	53	91.5	85.5
Shao[23]	China	2014	67/49	bDNA	NR	III-IV	G1-G3	serum	344.4 μg /L	56	8	11	41	83.9	84.2
Zachariah[24]	Switzerland	2008	21/36	RT-PCR	NR	NR	NR	plasma	2653GE/ml	16	11	5	25	74.0	69.0

No. of P/C = number of patients/ control; RT-PCR = real-time quantitative PCR; bDNA = branched DNA; TP = true positive; FP = false positive; FN = false negative; TN = true negative. NR: not reported.

The quality assessment results of the eligible nine studies are shown in Table 2. In general, most studies had a moderate-high quality. Two major problems were found. One was that the patient selection in five studies augmented the risk of selection bias and applicability concerns due to the case-control study design [11, 16, 18, 19, 21]. The other was that the use of a blinding method for a reference standard was not mentioned in five studies [16, 19, 20, 22, 23], which may result in an unknown risk of performance bias in relevant articles.

**Table 2:** Quality assessment of the studies selected for the meta-analysis (QUADAS-2).

Study	Risk of Bias				Applicability Concerns		
	Patient Selection	IndexTest	ReferenceStandard	Flow and Timing	Patient Selection	IndexTest	ReferenceStandard
Capsize [16]	H	L	U	L	L	L	L
Chang [17]	U	U	L	U	U	L	L
Fu [18]	H	L	L	U	L	L	L
Gu [19]	H	L	U	U	U	L	L
Guan [20]	L	L	U	L	L	L	L
Holdenrieder [21]	H	U	L	L	L	L	L
Kamat [22]	H	+	U	L	L	L	L
Shao [23]	L	L	U	U	L	L	L
Zachariah [24]	L	L	L	U	L	L	L

L, low risk; H, high risk; U, unclear risk.

### Diagnostic accuracy

**Fig 2:**
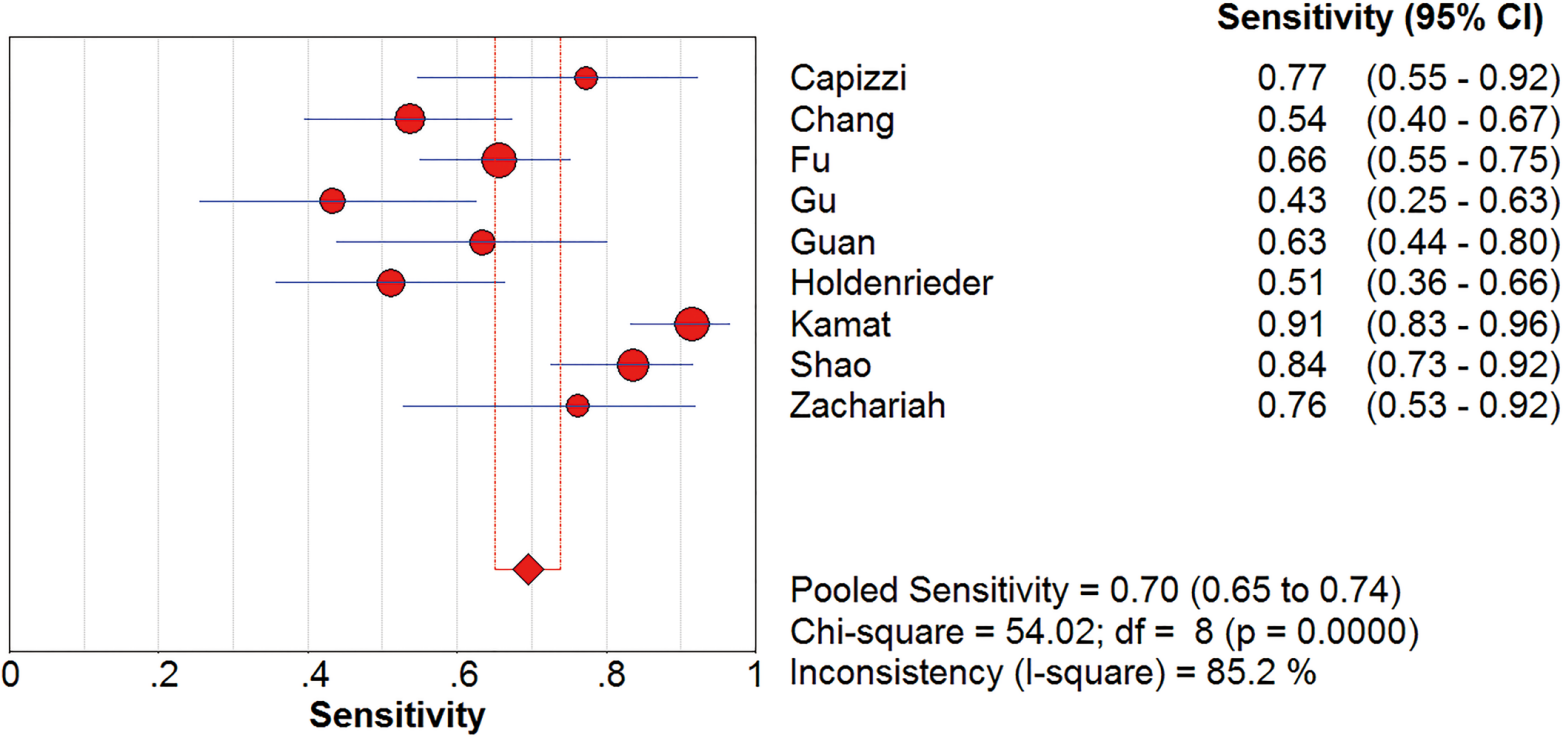
Forest plot of estimated sensitivity for quantitative analysis of circulating cell free DNA in the diagnosis of ovarian cancer. CI = confidence interval.

**Fig 3:**
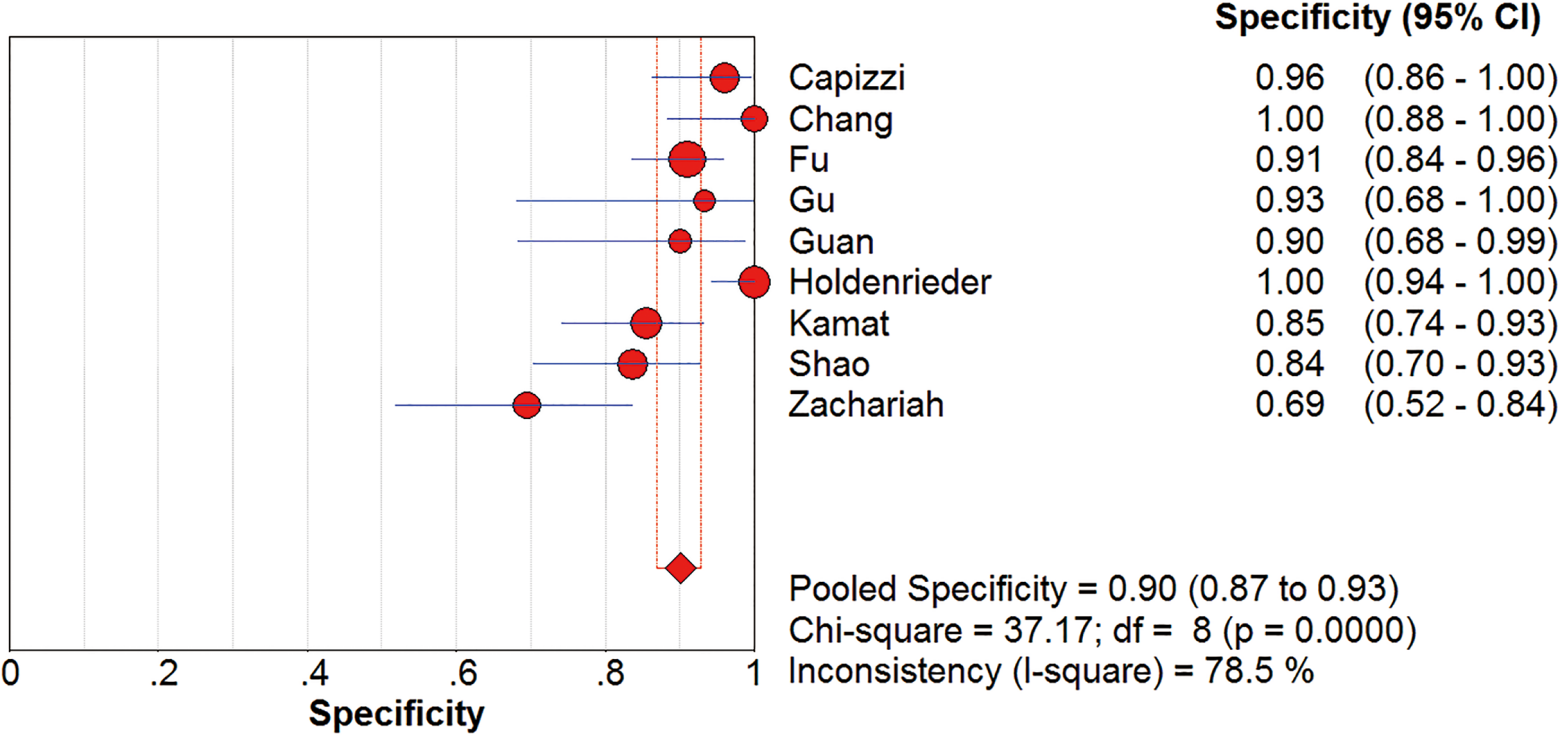
Forest plot of estimated specificity for quantitative analysis of circulating cell free DNA in the diagnosis of ovarian cancer. CI = confidence interval.

**Fig 4:**
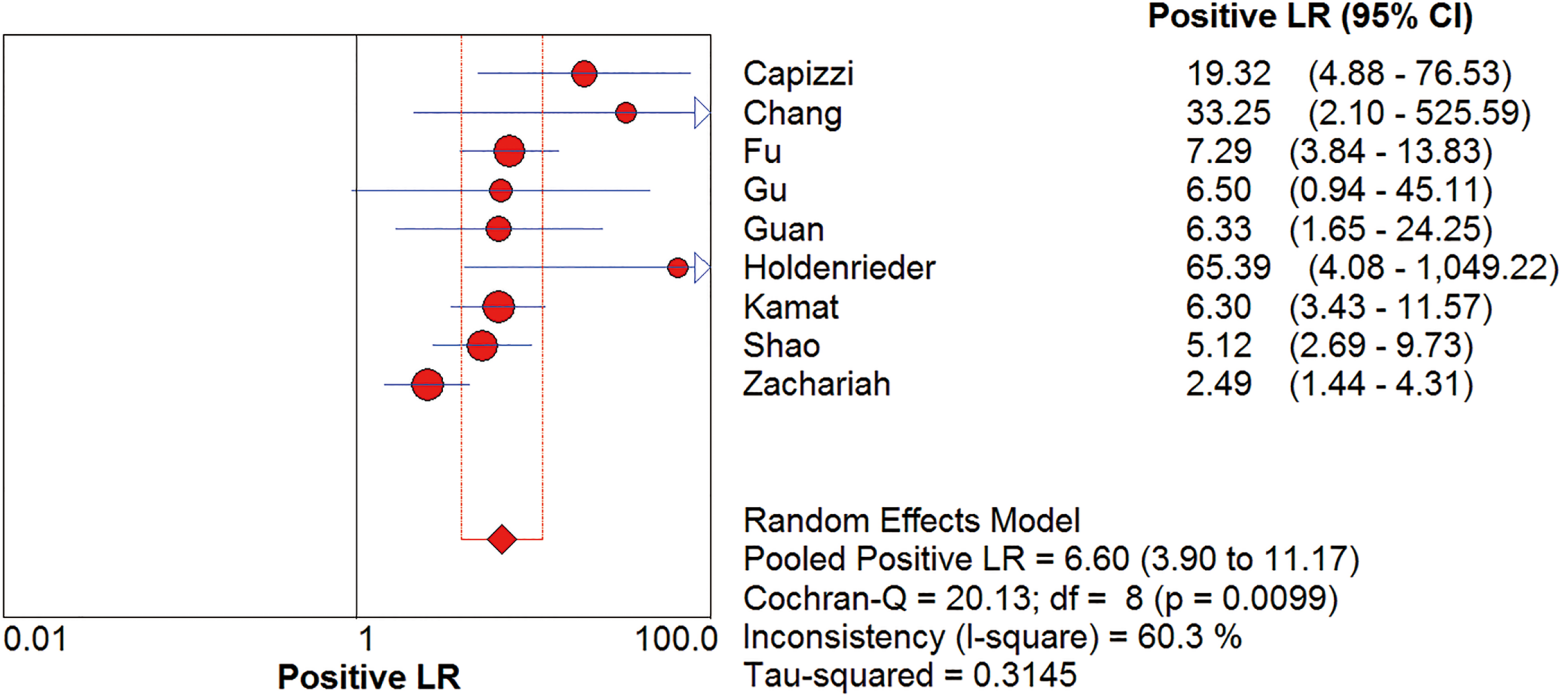
Forest plot of estimated PLR for quantitative analysis of circulating cell free DNA in the diagnosis of ovarian cancer.

**Fig 5:**
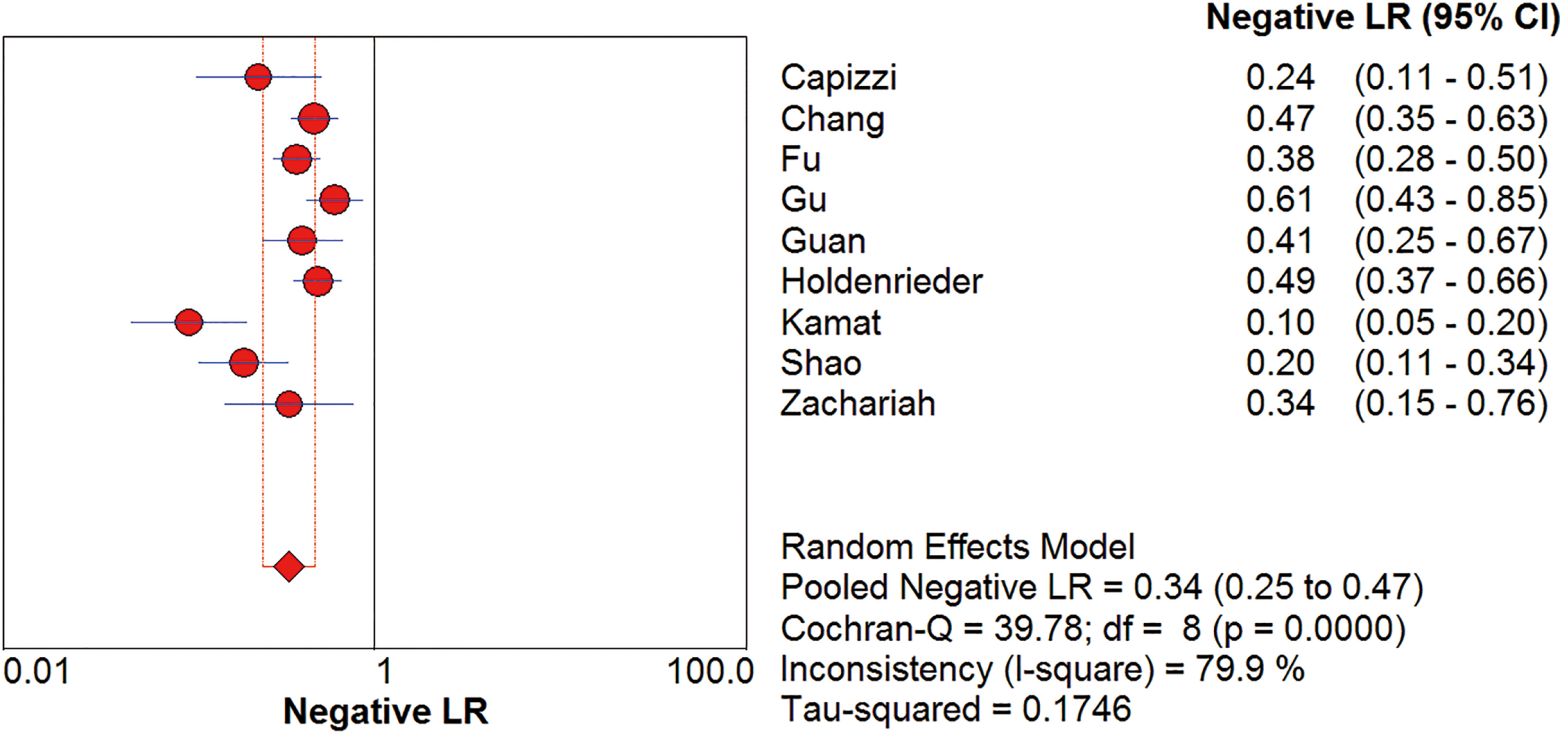
Forest plot of estimated NLR for quantitative analysis of circulating cell free DNA in the diagnosis of ovarian cancer.

**Fig 6:**
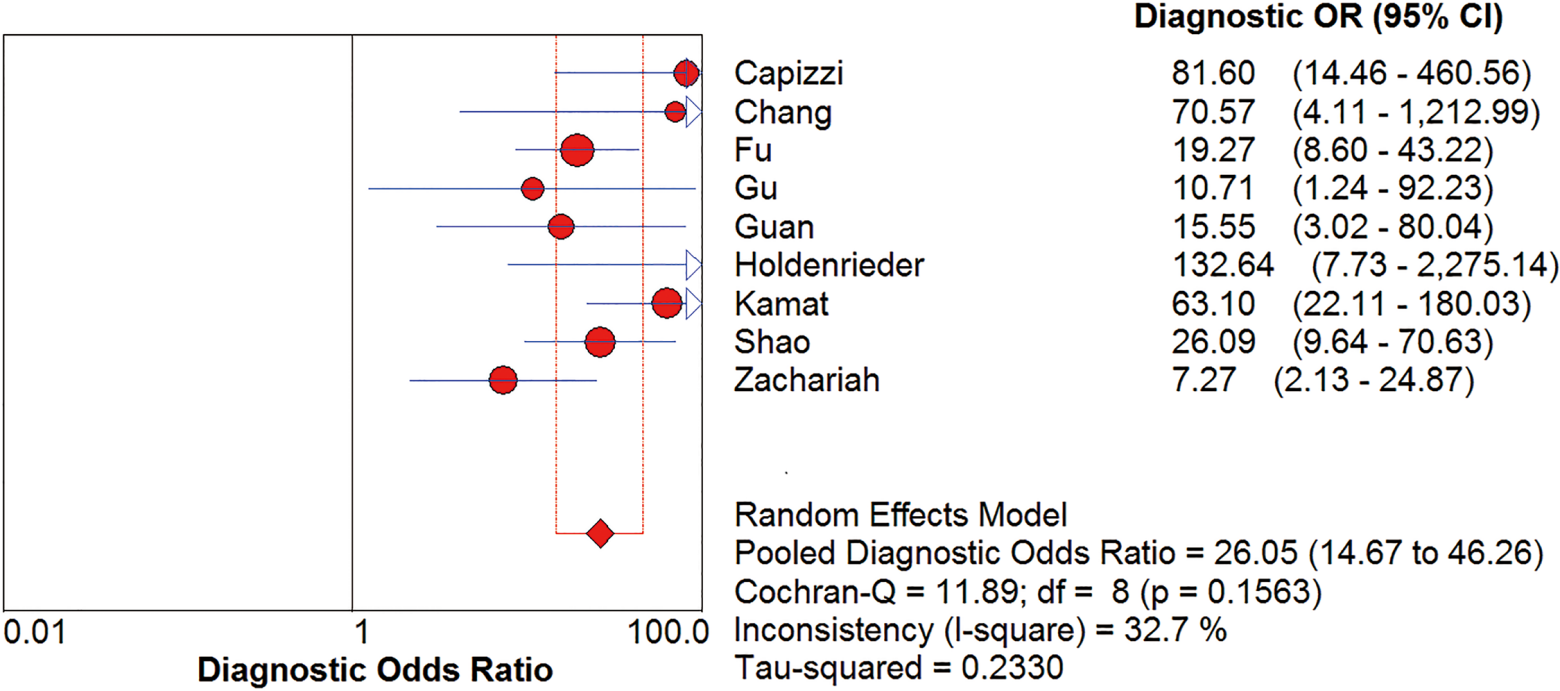
Forest plot of estimated DOR for quantitative analysis of circulating cell free DNA in the diagnosis of ovarian cancer.

**Fig 7:**
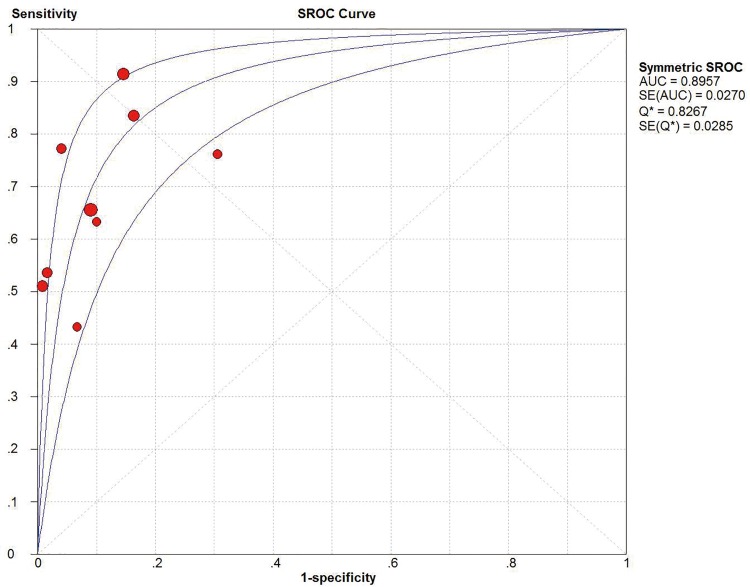
The SROC curve for quantitative analysis of circulating cell free DNA in the diagnosis of ovarian cancer.

*I*^2^ test showed obvious inter-study heterogeneity (*I*^2^ = 85.2% for sensitivity and *I*^*2*^ = 78.5% for specificity), suggesting high levels of heterogeneity in the nine studies. The threshold effect was the major cause of heterogeneity. In the meta-analyses, the Spearman correction coefficient was 0.633 (*P*>0.05), confirming that the threshold effect was not significant and the heterogeneity caused by other reasons. Forest plots of the sensitivity and specificity for circulating cfDNA in ovarian cancer diagnosis are shown in Figs 2 and 3. The pooled sensitivity and specificity were 0.70 (95% CI 0.65–0.74) (Fig 2) and 0.90 (95% CI, 0.87–0.93) (Fig 3), respectively. In addition, the pooled PLR was 6.60 (95% CI, 3.90–11.17) (Fig 4), NLR was 0.34 (95% CI, 0.25–0.47) (Fig 5) and diagnostic odds ratio was 26.05 (95% CI, 14.67–46.26) (Fig 6). The SROC curve for the included studies is shown in Fig 7. The AUC was 0.89 (95% CI 0.83–0.95), indicating a relatively high accuracy of quantitative analysis of circulating cfDNA for ovarian cancer diagnosis.

Subgroup analyses were conducted for different subtypes, which included participants (Asia or Caucasian), specimen types (plasma or serum) and sample size (sample size≥100 or sample size<100). We found that studies on Asian populations group had a poor overall accuracy as compared with that of those on Caucasian populations, with sensitivity of 0.67 versus 0.74, specificity of 0.89 versus 0.91, PLR of 6.15 versus 9.51, NLR of 0.38 versus 0.30, DOR of 19.89 versus 42.38 and AUC of 0.90 versus 0.91, respectively. In addition, the subgroups based on sample size suggested that larger sample size groups were more accurate in detecting ovarian cancer than smaller sample size group in a sensitivity of 0.76 versus 0.62, specificity of 0.90 versus 0.89, PLR of 6.49 versus 7.54, NLR of 0.26 versus 0.43, DOR of 32.25 versus 19.21 and AUC of 0.91 versus 0.82, which reflect a higher potential diagnostic value of larger sample size. Furthermore, we also found that plasma-based assays showed a higher level of sensitivity (0.72 versus 0.65) but lower level of specificity (0.89 versus 0.93) and AUC (0.89 versus 0.90) as compared with serum-based assays, which indicate that the best source for reliable cfDNA detection cannot be determined by current evidence. The pooled data such as sensitivity, specificity, PLR, NLR, DOR, and AUC for each subgroup are shown in Table 3.

**Table 3:** Results of the subgroup analyses performed to identify potential sources of heterogeneity.

Variables	No of studies	SEN (95% CI)	SPE (95% CI)	PLR (95% CI)	NLR (95% CI)	DOR (95% CI)	AUC
Overall	9	0.70 (0.65–0.74)	0.90 (0.87–0.93)	6.60 (3.90–11.17)	0.34 (0.25–0.47)	26.05 (14.67–46.26)	0.89
**Participants**							
Asia	4	0.67 (0.61–0.74)	0.89 (0.83–0.93)	6.15 (4.04–9.35)	0.38 (0.24–0.58)	19.89(11.30–35.00)	0.90
Caucasian	5	0.74 (0.65–0.72)	0.91 (0.86–0.94)	9.51 (3.03–29.89)	0.30 (0.18–0.53)	41.38(12.71–134.66)	0.91
**Specimen types**							
Plasma	6	0.72 (0.66–0.77)	0.89 (0.85–0.92)	6.53 (3.36–12.69)	0.31 (0.21–0.47)	26.32(12.10–57.24)	0.89
Serum	3	0.65 (0.56–0.73)	0.93 (0.87–0.97)	8.27 (2.27–30.09)	0.41 (0.22–0.73)	26.28(11.00–62.78)	0.90
**Sample size**							
Size≥100	4	0.75 (0.69–0.79)	0.90 (0.86–0.93)	6.49 (4.32–9.77)	0.26 (0.14–0.47)	32.25 (16.54–62.88)	0.91
Size<100	5	0.59 (0.51–0.67)	0.89 (0.83–0.93)	7.54 (2.26–25.18)	0.43 (0.32–0.59)	19.21(7.06–52.25)	0.82

SEN = Sensitivity; SPE = Specificity; TP = true positive; FP = false positive; FN = false negative; TN = true negative. NR: not reported.

### Meta-regression analysis for heterogeneity

To explore possible sources of the heterogeneity across these nine studies, we performed a meta-regression analysis to assess covariates used in the studies. The following specific variables were evaluated for their impacts on heterogeneity: “Publication year” (Year), “Study location” (Region: Asia or not), “Specimen types” (Sample: Plasma or Serum), “Study design” (Design: prospective or not) and “Assay methods” (Methods). However, none of these factors showed any definite influence on heterogeneity (Table 4). We also noticed that differences in studies with or without “Patient selection”, “Index Test”, “Reference Standard” and “Flow and Timing” (four key domains in QUADAS-2) did not cause statistically significant differences among studies (Table 4), indicating that the study design did not substantially affect the diagnostic accuracy. Heterogeneity may have arisen due to other reasons, such as the number of included patients, age, tumor type, tumor size, metastasis, TNM staging and differences in the operating protocol, which could not be analyzed in the present study due to partial loss of the data or unrecognizable details.

**Table 4:** Results of the meta-regression performed to identify potential sources of heterogeneity.

Covariates	Coefficient	Std.Err.	RDOR (95% *CI*)	*p* value
Publication year	-0.310	0.1355	0.73(0.53–1.02)	0.0624
Study location	-0.335	0.4091	0.72(0.26–1.95)	0.4091
Sample	-0.057	0.7916	0.94(0.14–6.55)	0.9451
Assay method	0.088	0.3080	1.09(0.51–2.32)	0.7853
Study design	-0.654	0.5567	0.52(0.13–2.03)	0.2849
Patient selection	-0.709	0.6478	0.49(0.10–2.40)	0.3155
Index Test	-1.671	1.5547	0.19(0.00–8.44)	0.3236
Reference Standard	-0.788	0.5489	0.45(0.12–1.74)	0.2013
Flow and Timing	1.094	0.4738	2.98(0.94–9.51)	0.0604

Std.Er, standard error; CI, confidence interval.

### Publication bias estimate

**Fig 8:**
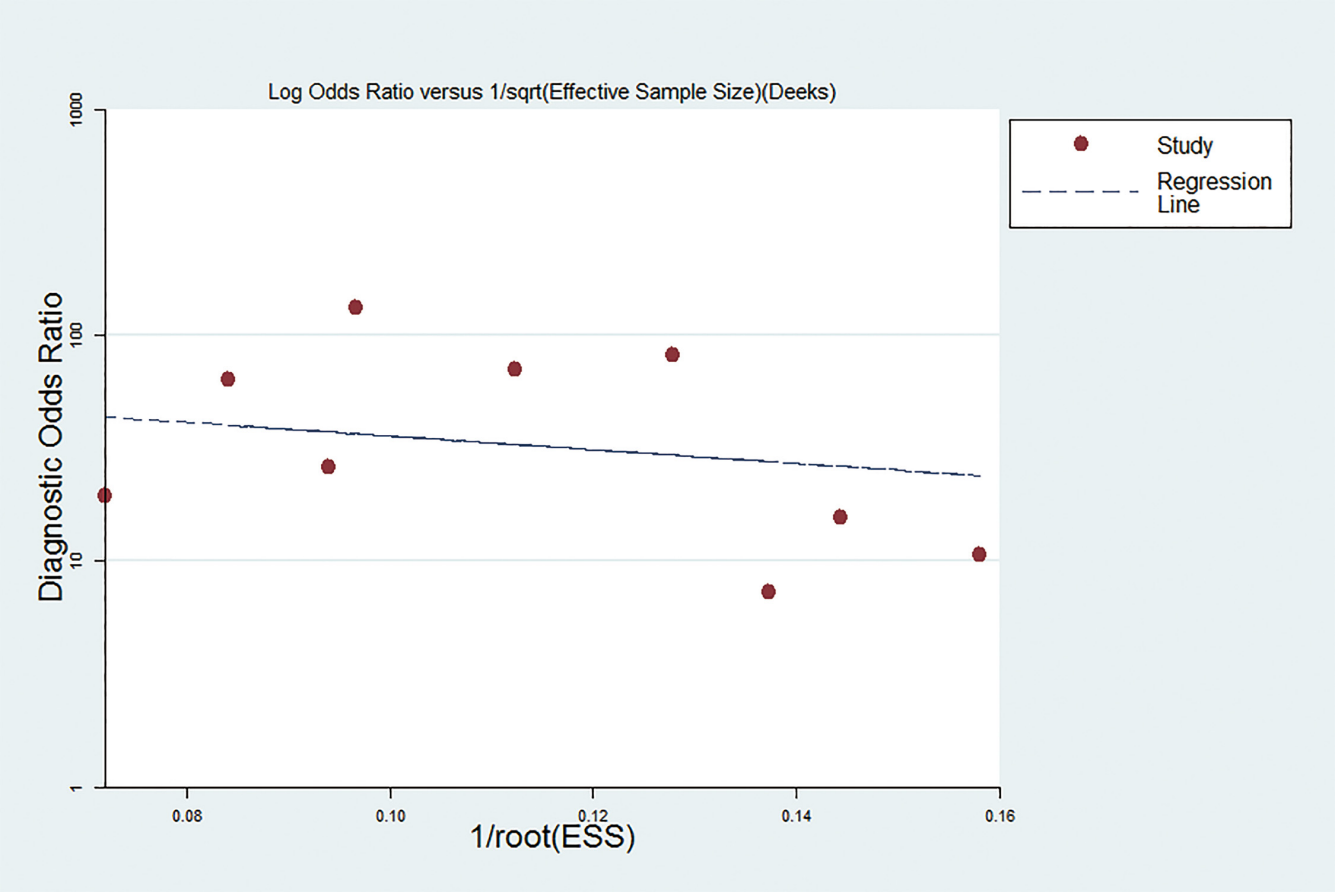
The Deeks’ funnel plot for the assessment of potential publication bias of the included studies.

Publication bias is assessed visually by using a scatter plot of the inverse of the square root of the effective sample size (1/ESS1/2) versus the diagnostic log odds ratio (lnDOR), which should have a symmetrical funnel shape when publication bias is absent[34]. In this meta-analysis, Deeks’ funnel plot asymmetry test was used to evaluate publication bias of the included studies. The slope coefficient was associated with a P value of 0.142, suggesting an existing low likelihood of publication bias in this met analysis. The Deeks’ funnel plot for the assessment of potential publication bias of the included studies is shown in Fig 8.

## Discussion

The existence of cfDNA in human blood was initially described by Mandel and Metais [35]. Currently, multiple studies have demonstrated that the serum or plasma level of cfDNA in cancer patients is generally higher than that in healthy individuals [15–24]. There has been great interest in the potential use of circulating cfDNA for non-invasive diagnosis of cancer [36]. Specifically, two previous meta-analyses have reported the diagnostic accuracy of quantitative analysis of circulating DNA is at least the same as the conventional biomarkers for the diagnosis of lung cancer [37] and hepatocellular carcinoma [38], One recently published closely related meta-analysis reported that the high levels of cfDNA were associated with the worse survival in solid tumors[39]. For ovarian cancer diagnosis, screening biomarkers have been widely studied, but few have satisfactory performance for clinical applications [4, 5]. Ovarian cancer remains a poor prognostic disease as vague or nonspecific symptoms lead to delayed diagnosis [8]. Thus, there is an urgent need to improve early detection methods and identify new diagnostic biomarkers for the disease [4, 5]. Quantitative analysis of circulating cfDNA for ovarian cancer diagnosis has attracted increasing attention, leading to dozens of studies. However, the results of those studies are varied and have not been systematically evaluated [16–24]. Hence, for the first time, we performed this comprehensive meta-analysis to integrate all related publications and evaluate the accuracy of circulating cfDNA as a diagnostic biomarker for ovarian cancer.

The pooled sensitivity and specificity of the circulating cfDNA assay were 0.70 (95% CI 0.65–0.74) and 0.90 (95% CI, 0.87–0.93), respectively, indicating quantitative analysis of cfDNA has poor sensitivity but acceptable specificity for diagnosis of ovarian cancer. Likelihood ratios (LRs) are metrics that reflect the verity of sensitivity and specificity: LRs of >10 or <0.1 generate large and often conclusive shifts from pretest to posttest probability [40]. LRs are more clinically meaningful than SROC curve and DOR. In our study, the pooled PLR and NLR of the circulating cfDNA assay was 6.60 (95% CI, 3.90–11.17) and 0.34 (95% CI, 0.25–0.47), respectively. This result indicated that ovarian cancer patients have approximately seven times greater chance of being circulating cfDNA assay-positive compared with healthy controls, and an approximately 34% error rate would be present when the true negative was determined in the cfDNA assay-negative test. These results indicated that the unsatisfactory likelihood ratios obtained in meta-analysis may indicate poor robustness and accuracy. Moreover, a DOR of 1.0 indicates that a test does not discriminate between patients with the disorder and those without it [41]. The mean DOR in our study was 26.05 (95% CI, 14.67–46.26), indicating a relatively high level of overall accuracy. Notably, subgroup analyses were conducted for three different subtypes. We found that studies on Asian populations group had a poor overall accuracy as compared with that of those on Caucasian populations with lower level of sensitivity, specificity, DOR and AUC. Meanwhile, our subgroup analysis suggested that larger sample size groups were more accurate in detecting ovarian cancer than smaller sample size groups were. As for specimen types, we also found that plasma-based assays showed a higher level of sensitivity but lower level of specificity and AUC as compared with those of serum-based assays, indicating that the best source for reliable cfDNA detection cannot be determined by current evidence. Further large-size studies focusing on differential racial background, sample size and specimen types are required to confirm these findings.

During the past few decades, a number of circulating markers have been proposed for the early detection of ovarian cancer, and two of the commonly used biomarkers (carbohydrate antigen 125 [CA125] and human epididymis protein 4 [HE4]) have been approved by the US FDA for the risk assessment or management of ovarian cancer [42]. Recent meta-analysis reported the diagnostic performance of CA125 and HE4 in ovarian cancer. According to these studies, the AUC of SROC curve for CA125 and HE4 are 0.84–0.87 [43–45] and 0.87–0.92 [44–47], respectively. In our meta-analysis, the AUC of SROC for cfDNA was 0.89 (95% CI 0.83–0.95), indicating a relatively high accuracy of circulating cfDNA for ovarian cancer diagnosis. Regrettably, rare studies directly compare the diagnostic value of cfDNA with other conventional markers, thus our study could not elucidate whether alone or combined circulating cfDNA improves the diagnostic accuracy of commonly used serum tumor markers for ovarian cancer screen.

Heterogeneity is an important issue in meta-analysis. In the present meta-analysis, significant heterogeneity was detected among the included studies by the *Q*-test and *I*^*2*^ statistic of inconsistency analysis. The threshold effect is a primary cause of heterogeneity in accuracy test studies. However, the spearman correction coefficient of the current study (0.633, *P*>0.05) indicated that the heterogeneity was not caused by the threshold effect and the heterogeneity caused by other reasons. In order to explore the potential source of heterogeneity, we investigated the characteristics of included studies such as publication year, study location, sample type, study design and assay methods using subgroup analyses and meta-regression, but no covariables were found to contribute to the heterogeneity. We also noticed the differences between studies with or without “Patient selection”, “Index Test”, “Reference Standard” and “Flow and Timing” (four key domains in QUADAS-2). However, subgroup analyses and meta-regression analysis revealed that differences in these factors did not significantly affect heterogeneity, indicating that the study design did not substantially affect the diagnostic accuracy and the influencing factors are unclear. In addition, the publication bias was not significant, indicating that the results of our meta-analysis are reliable.

The current meta-analysis does have some limitations. First, circulating cfDNA is a newly discovered tumor biomarker, and the number of studies that can be included in the meta-analysis is small, which leads to poor robustness of some of the pooled analysis results. This can be improved when more studies are available. In addition, four of these nine eligible studies were from China and only English or Chinese language studies were included, which may yield selection bias for the specifically studied population or language. Moreover, heterogeneity existed between different studies but the sources of heterogeneity could not be identified by subgroup analyses and meta-regression analysis.

## Conclusions

In conclusion, current evidence suggests that the diagnostic accuracy of circulating cfDNA has unsatisfactory sensitivity but acceptable specificity for diagnosis of ovarian cancer. Further large-scale prospective studies are required to validate the potential applicability of using circulating cfDNA alone or in combination conventional markers as ovarian cancer diagnostic biomarker and explore potential factors that may influence the accuracy of circulating cfDNA for ovarian cancer diagnosis.

## Supporting Information

S1 Appendix PRISMA Checklist. PRISMA 2009 Checklist.
(DOC)
